# Evaluation of a Multidisciplinary Bachelor Course on Pain with Autonomy-Supportive Teaching Strategies through the Lens of Self-Determination Theory

**DOI:** 10.3390/pharmacy9010066

**Published:** 2021-03-23

**Authors:** Adriana H. van Houwelingen, Rashmi A. Kusurkar, Ferdi Engels

**Affiliations:** 1 Division of Pharmacology, Department of Pharmaceutical Sciences, Science Faculty, Utrecht University, 3584 GC Utrecht, The Netherlands; G.M.H.Engels@uu.nl; 2 Amsterdam UMC, Research in Education, Faculty of Medicine, Vrije Universiteit Amsterdam, 1081 HV Amsterdam, The Netherlands; R.Kusurkar@amsterdamumc.nl; 3 LEARN! Research Institute for Learning and Education, Faculty of Psychology and Education, Vrije Universiteit Amsterdam, 1081 HV Amsterdam, The Netherlands

**Keywords:** autonomy, multidisciplinary course, pain, students’ perception, undergraduate, bachelor

## Abstract

To stimulate learners’ autonomy, autonomy-supportive teaching strategies were included in the design of a multidisciplinary elective course on pain. During this course, students explored pain from different disciplinary angles, i.e., from biomedical, psychological, arts, philosophical, and anthropological perspectives. In the course, autonomy was stimulated by giving students freedom of choice, especially in their final assignments. The aim of this study was to explore students’ freedom of choice and students’ perceptions of the multidisciplinary course on pain, particularly students’ perception of autonomy in the light of self-determination theory (SDT). To address the aim of this study, a mixed methods approach was used. Directed content analysis was conducted on a reflective part of the final individual assignment and was used to find categories fitting within SDT and also outside it. In addition to this, the diversity of topics as well as different disciplines present in the final individual assignments was explored to demonstrate students’ freedom of choice. This study shows that the course setup supported students’ autonomy and relatedness and stimulated students’ interest in and relevance to pain. Moreover, it stimulated students’ freedom of choice and stimulated curiosity towards disciplines such as arts and philosophy. Therefore, it can be concluded that we successfully developed a multidisciplinary course on pain in which students are exposed to different autonomy-supportive teaching strategies based on the SDT framework.

## 1. Introduction

Learners’ autonomy was originally defined by Holec (1979) as “the ability to take charge of one’s own learning” and has been extensively studied since then, especially in the field of language teaching and learning [1]. Learners’ autonomy, as defined by Holec, involves motivation and engagement for what needs to be learned as well as skill development of self-regulation in students, enabling them to take responsibility for their own learning. In the current times, higher education curricula are being increasingly designed and developed to provide learners with autonomy for their learning, but the extent to which these attempts are successful is unknown. Moreover, it has been reported that there is discordance between the level of autonomy that the teachers in health professions education think they provide and the level of autonomy that the students perceive [2]. The aim of this study was therefore to explore students’ freedom of choice and students’ perceptions of the multidisciplinary course on pain, particularly students’ perception of autonomy. 

Learners’ autonomy can be studied starting from different motivational theories, among them is Ryan and Deci’s self-determination theory (SDT). Based on SDT, students’ intrinsic motivation and self-regulation can be stimulated by supporting three basic psychological needs: autonomy, competence, and relatedness [3]. Autonomy refers to an experience of freedom and choice enabling individuals to take responsibility in voluntary actions. Positive effects of autonomy-supportive activities were found on student motivation, engagement, and well-being [4,5,6,7]. Moreover, perceived autonomy support as well as intrinsic motivation was associated with higher student ratings and academic success [8,9]. These studies demonstrate the beneficial effects of incorporating autonomy-supportive activities in learning environments for both students and teaching staff.

To stimulate students’ autonomy, teachers can use autonomy-supportive teaching strategies that are based on three main principles: offering choices in learning, providing a rationale for learning, and organizing learning in accordance with the learners’ perspective and interests [3,10]. As demonstrated by Garcia and Pintrich, giving choices, even as little as decision making, positively influenced students’ intrinsic goal orientation, task value, and self-efficacy [11]. Stefanou et al. further explored the concept of providing choices in cognitive, procedural, and organizational features [12]. Cognitive choices include students looking for multiple solutions to a problem, bringing in and debating about own ideas, and setting intrinsic (life) goals for learning. Cognitive choices may enhance deep-level thinking about the topic at hand. Procedural choices include students choosing learning resources and materials, choosing how competence will be demonstrated, and discussing what they want to learn in the course. Procedural choices may encourage initial engagement with learning. Organizational choices include students choosing group members, choosing types of evaluation, and deciding the rules of engagement. Organizational choices may enhance well-being and comfort with the class. Altogether, by giving students choices, teachers can create opportunities for students to explore their own learning goals and needs within a learning environment, thereby stimulating students’ goal setting, engagement, and intrinsic motivation.

In many pharmacy curricula, providing learners with autonomy, and especially choices, seems to be at odds with the framework imposed by the educational program with respect to content and competencies that pharmacy students need to develop in order to become a pharmacist [13]. To support students’ autonomy, we sought to explore the limits of these constraints by developing an elective course on pain that possesses autonomy-supportive teaching elements and which goes beyond the traditional student-centered subject-focused teaching imposed by strict curricular guidelines defined by the pharmacy program. Pain as the overall topic for the course was chosen for two reasons. Firstly, pain is poorly represented in many health care-oriented curricula, such as pharmacy. Observations from studies in the UK, the USA, and Spain revealed that education on pain in health care curricula is limited and fragmented [14,15,16]. In most pharmacy curricula, the topic of pain and its treatment are often discussed as part of the pathophysiology of diseases and ailments and receive relatively little attention. Secondly, the phenomenon of pain is far more complex and also involves the patients’ experiences, perceptions, and attitudes. Learning about these aspects will benefit mutual understanding between health care professionals and patients, is important for patient satisfaction, and may improve clinical outcomes [17,18].

To stimulate students’ intrinsic motivation, autonomy-supportive teaching strategies are useful and easy to include in the design of any course, whether it has a large classroom setup or small group meetings [19]. In the design of the elective course on pain, different autonomy-supportive teaching strategies were used. Firstly, students had to set their own intrinsic goals and apply them in the context of learning. Secondly, pharmacy students had to take their responsibility in course activities, group discussions, and groupwork as most of these activities were unsupervised. Thirdly, the role of the teacher in this course was to be a coach who mostly facilitates students’ learning processes. Lastly, pharmacy students could choose their own topic for their final individual assignment and group assignment. 

As already described, giving students choices in our course on pain can be important for their motivation and engagement in the topic. However, giving pharmacy students much freedom of choice with respect to what they learn seems rather at odds with common practice in many curricula, not only from an organizational perspective but also from students’ perspective. Therefore, we were interested in students’ perceptions of autonomy, especially within this unique course setup where students have to define their own learning goals within a pharmacy curriculum with strict curricular guidelines. Therefore, the aim of this study was to evaluate and explore students’ perceptions of the multidisciplinary course on pain, particularly students’ perception of autonomy in the light of SDT. Moreover, students’ freedom of choice and curiosity towards other disciplines were investigated. A further goal of this article is to inspire other educators with respect to multidisciplinary contents and approaches of this course on pain.

## 2. Methods

### 2.1. Course Setting and Participants

The elective course was hosted by the Department of Pharmaceutical Sciences and was part of the bachelor pharmacy curriculum. The pharmacy curriculum at Utrecht University consists of a 3-year bachelor and a 3-year master program. Both programs have been developed according to national agreed standards with respect to the disciplinary content and competencies, which optimally prepares the students for either a career as a pharmacist or a drug researcher. 

At Utrecht University, an academic year consists of two semesters. Each semester consists of two consecutive time periods of 10 weeks. In the 10 weeks’ time period, two courses are scheduled in parallel. Each course has a study load of 200 h, yielding 7.5 credits according to the European Credit Transfer System (ECTS).

From August 2015 till August 2018, the elective course on pain was scheduled in the fourth period of the academic year as an advanced-level course. The enrollment was limited to 40 students to allow personal interactions not only amongst students but also between students and guest lecturers and students and the main teacher of the course. 

Participants were predominantly pharmacy students, although students from biomedical sciences, psychology, social sciences, artificial intelligence, liberal arts and sciences, anthropology, medicine, biology, chemistry, and science communication participated in this course. 

### 2.2. Course Description and Its Activities

**Figure 1:**
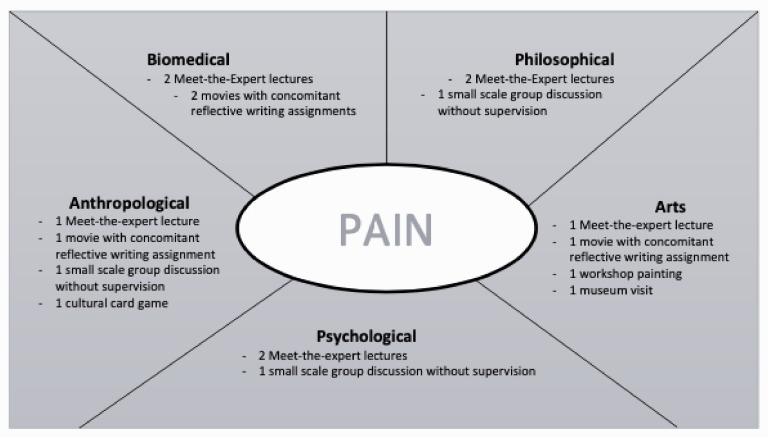
Overview of learning activities during the 10-week course.

During the course, students explored the phenomenon of pain from five different disciplinary perspectives, in five consecutive 2-week blocks. These perspectives were: biomedical aspects of pain, philosophy of pain, arts and pain, psychology of pain, and anthropology of pain (see Figure 1).

In general, each of the five perspectives was introduced by an expert lecturer in the respective field and accompanied by introductory reading material. Although students had access to introductory reading material, they were encouraged to gain deeper knowledge by searching for literature and building an understanding of a specific topic of interest since they had defined their own learning goals at the beginning of the course. The small group meetings were built around introductory reading material and were not supervised by the main teacher (A.H.v.H). However, the teacher was available at distance and on demand. During these meetings, students were in control of their learning and had the opportunity to share and discuss their opinions and understanding with their peers. Documentary films were used to arouse students’ interest and to stimulate students’ perspective taking. In addition to watching these documentary films, students were instructed to write a reflective essay for each film. These reflective essays supported students in their preparation for their final individual assignment. 

#### 2.2.1. Biomedical Perspective of Pain

As an introduction to this perspective, and in general for the whole course, a Dutch documentary film by Suzanne Raes (©Pijn, 2005, by Lemming Film BV and VPRO, Hilversum, The Netherlands) was shown. Raes followed six patients whose lives are controlled by pain. In addition to that, a documentary film by Melody Gilbert (©Frozen Feet Films, 2005, St. Paul, MN, USA) explored the opposite, describing three children with congenital insensitivity to pain. Students watched both documentary films in class, allowing them to share ideas and experiences while watching. Besides presenting factual knowledge, these documentary films also visualized the feelings and mood of the patients and their relatives. Biomedical aspects of pain were discussed in two lectures, one presented by a general pharmacologist and the other by an anesthesiologist. 

#### 2.2.2. Philosophical Perspective of Pain

Philosophical considerations of the phenomenon of pain were presented in a variety of ways. After introductory reading material answering the basic question, “What is philosophy?”, a set of 2-h lectures by a philosopher took the students along the path of two worlds theory and body–mind dualism and applied these concepts to the phenomenon of pain. Original writings by Descartes and Ryle were compulsory reading assignments, which culminated in a small group discussion in which students discussed philosophical statements on pain with their peers. 

#### 2.2.3. Pain and Arts

The subject of pain and art was introduced by the philosophy lecturer who discussed the representability of pain in art. Students were taken along the questions whether pain may, must, and can be expressed in art. A documentary film by Amy Stechler (©The life and Times of Frida Kahlo, 2005, Daylight Films and WETA, Arlington, VA, USA) on the Mexican artist Frida Kahlo was shown, demonstrating how physical and mental pain can influence a person’s life and how Kahlo used art to cope with her pain. Further activities concerning this subject focused on experiencing pain in art. In small groups, students visited a museum of their choice and had to work on an assignment which consisted of choosing two pieces of art that they associate with pain. Students shared their insights about these artworks and the artistic trends they had been studying by presenting their findings to their fellow students. Finally, a workshop was organized for students to make their own painting about pain. The workshop was led by an artist who had no connections with the university itself. 

Both activities (museum visit and painting clinic) were scheduled for students not only to experience the effect art and artistic expression could have on pain relief but also to experience the differences in how individuals could express pain. This could help students to understand differences in pain and pain expression by patients. 

#### 2.2.4. Psychological Perspective of Pain

The psychology of pain was introduced by a clinical psychologist who discussed psychological interventions in otherwise untreatable pain. In addition to the psychologist, an episode of a Dutch television series (©Katja’s Bodyscan, 2016, KRO-NCRV, Hilversum, The Nederlands) in which a well-known TV celebrity employs her body and mind for the benefit of science and that deals with the psychology of pain was shown. Subsequently, students investigated the role of emotions in pain behavior by reading compulsory literature and discussing and mind mapping their outcomes in a small group meeting. 

#### 2.2.5. Anthropological Perspective of Pain

Cultural differences in how pain is perceived, and sometimes actually appreciated, were shown through the documentary film “Sacred Pain” (©Sacred Pain, 2003, National Geographic, Washington, DC, USA). This film shows the diversity of reactions of people to pain. It often leads to very strong emotions. Through the combination of, on the one hand, eliciting emotions among the students when they observe how people inflict pain on themselves and, on the other hand, providing information on cultural differences of how people approach pain, students learned about diversity in which meaning making is very powerful. Besides the documentary film, students also had to study literature about cultural differences with respect to perception of pain. During a small classroom meeting, students had to discuss their own trues and values on pain. Both the documentary and the small classroom setting provided students with a deeper understanding of how other people face pain from their culture or perspective.

#### 2.2.6. Course Assignments

Students had to work towards two final assignments, a group assignment and an individual assignment. The description of the group assignment was as follows: bring the phenomenon of pain in a ludicrous way to the spotlight, and organize a meeting in which pain can be seen, felt, tasted, heard, smelled, etc. The individual assignment consisted of two parts: an essay on a topic they relate to pain and a personal reflection on the course and the course setup. In the essay, at least three out of five course perspectives should be present to meet the assignment criteria. Topics for the essay should relate to pain and could consist of a song, a piece of art, a movie, or even a personal story. This essay was assessed with a rubric on originality (40%), the integration of the different course perspectives (30%), quality of students’ own interpretation on the topic (20%), and by the quality of writing (10%). The rubric was available for the students from the beginning of the course. In the second part of the individual assignment, the overall course reflection, students were asked to reflect on the course setup which provided information on students’ perception of the course. This reflection was compulsory but not graded. The students were asked to use the questions included in Table 1 as a guide for their writing.

### 2.3. Measures

To investigate students’ perception of the elective course on pain, a mixed methods approach was used. Both qualitative and quantitative data were analyzed from three consecutive academic years (2015–2016, 2016–2017, and 2017–2018). 

Qualitative data were collected from the reflective part of the individual assignment. Content analysis was conducted on the qualitative data. Content analysis is a research method in which the content of text data can be subjectively interpreted through the process of coding and identifying categories or patterns [20]. Within this, a directed content analysis approach was employed in which researchers use initial key concepts or codes based on an existing theory (in this study, SDT) as initial coding categories. Data from the whole first cohort (2015–2016) and randomly selected participants from 2016–2017 and 2017–2018 were analyzed until data sufficiency was reached. Data sufficiency was reached when enough data were analyzed to answer the research questions and no new codes/categories were generated on analyzing further data. For that, R.A.K. first read through all the data to familiarize herself with the content. In an iterative process, R.A.K. performed open coding of the data. In the second step, R.A.K. explored if the open codes fitted into the SDT categories of autonomy, competence, and relatedness. Any codes that did not fit into the existing categories were grouped into new categories. A.H.v.H checked the coding of every 10th participant. Discrepancies in coding between R.A.K. and A.H.v.H. were resolved through discussion and consensus. Qualitative data from each participant were not coded by two researchers independently but only by one coder. This is in line with Varpio et al., who argued against quantifying the rigor of qualitative data analysis [21]. It is not necessary that two coders code all qualitative data independently. Asking a second coder to code a few texts is an adequate way of ensuring rigor in qualitative research. 

The essay part of the individual assignments was analyzed for differences in topics to obtain an idea of students’ freedom of choice. In addition to this, the different course perspectives (biomedical, psychological, artistic, anthropological, or philosophical) were counted to explore students’ curiosity towards other disciplines. These quantitative data were expressed as frequency or percentage.

This study was approved by the faculty’s ethical committee of the Utrecht University (Bèta D-19259). 

## 3. Results

### 3.1. Students’ Perceptions on Autonomy, Relatedness, Interest, and Relevance

In most cases, the reflective part of the individual assignment consisted of a one-page written reflection of students’ overall perception of the course. Students elaborated on the course content, guest lecturers, the unsupervised small group meetings, and whether or not they liked the course setup. Moreover, they also provided suggestions for improvement. Not all students rated the course on a scale from 1 to 10, but these students provided expressions in wordings such as nice, inspiring, very interesting, and innovative.

For qualitative analyses, all 33 reflective parts of the individual writing assignment from cohort 2015–2016 (code numbers #001–#033), 4 essays from cohort 2016–2017 (code numbers #034, #041–#043), and 10 essays from cohort 2017–2018 (code numbers #097–#106) were analyzed by exploration of the SDT categories autonomy, competence, and relatedness. Data sufficiency was reached after analyzing the data of 47 essays. The following categories were generated: interest, autonomy, relatedness, and relevance. The description of the categories is as follows (see Table 2 for examples of quotations for each code within the categories):

- Interest (43 out of 47 essays)—Interest meant that students found the content or process of the course interesting. Within interest, we found the codes: *Novelty in Design and Content*, *Interesting Content*, *New Way of Thinking*, *Challenge,* and *Negative on Challenge*. Students found the course refreshing as they were experiencing such content and process for the first time in their curriculum.
  Moreover, it brought a new way of thinking and challenged the students by pushing them out of their comfort zones. Thus, this course was able to generate interest among students. Some students did find the recommended literature too challenging. In a minority of the essays (4 out of 47 essays), interest was not present.
- Autonomy (47 out of 47 essays)—Autonomy was mainly experienced through the openness of the course and was present in all essays. The following codes made up the theme of autonomy: *Own ideas, Own Structure, Own Initiative, Autonomous Learning, Active Participation, Personal Development,* and *Negative Choice*. Most of the students were happy with the opportunity to contribute their own ideas and structure regarding the content and the process of learning, and there was great room for taking their own initiative. They were able to also decide how deep they wanted to go into the topic depending on their interest. Thus, they were autonomous in their learning. Active participation was encouraged, the teacher was in the background, and there were avenues for developing personally. A minority of the students (3 out of 47 essays) did not appreciate the openness. These students wanted less autonomy and more teacher guidance. These students experienced autonomy as too far out of their comfort zone and/or they had the feeling that they have could learned more when the group meetings were supervised by the teacher. One aspect that worked against autonomy was that the students were pre-assigned to groups and did not get a chance to choose their group members themselves. This was seen as a problem only when fellow students did not collaborate well within the group.
- Relatedness (21 out of 47 essays)—Relatedness meant feeling part of a group. The following codes were included in this theme: *Learning from Each Other, Getting to Know Colleagues, Good Learning Environment,* and *Negative Collaboration*. The students valued learning from each other, especially different perspectives. They enjoyed getting to know colleagues through the course activities, which also helped them to work in a collaborative way. The learning environment was experienced as good in which students could freely express themselves. Collaboration was experienced negatively when fellow students in the group did not prepare or contribute enough to the group effort.
- Relevance (23 out of 47)—Relevance meant that students found the course relevant for their future. The codes that were grouped under this theme were: *Learning Other Perspectives*, and *For Practice.* About half of the students mentioned that they found looking at pain from different perspectives very interesting, important, and/or relevant for their future practice as pharmacists.

### 3.2. Students’ Freedom of Choice

In the elective course, students’ autonomy was stimulated in many different ways, one of them being freedom of choice. Since students could choose their topic of interest for their individual assignment, the topics of the final essays were very diverse, as can be seen in Table 3. The diversity of topics shows that students are able to relate pain to different topics.

As can be observed from Table 3, song texts were the most dominant topics of 2017–2018 while (World Press) photos and personal stories were most dominant in 2015–2016 and 2016–2017, respectively. Moreover, students related pain not only to diseases but also to historical events, religion, and historical figures. World Press photos (World Press Photo House, Amsterdam, The Nederlands) are photos that have national and/or international impact on society.

### 3.3. Students’ Curiosity towards Other Disciplines

**Figure 2:**
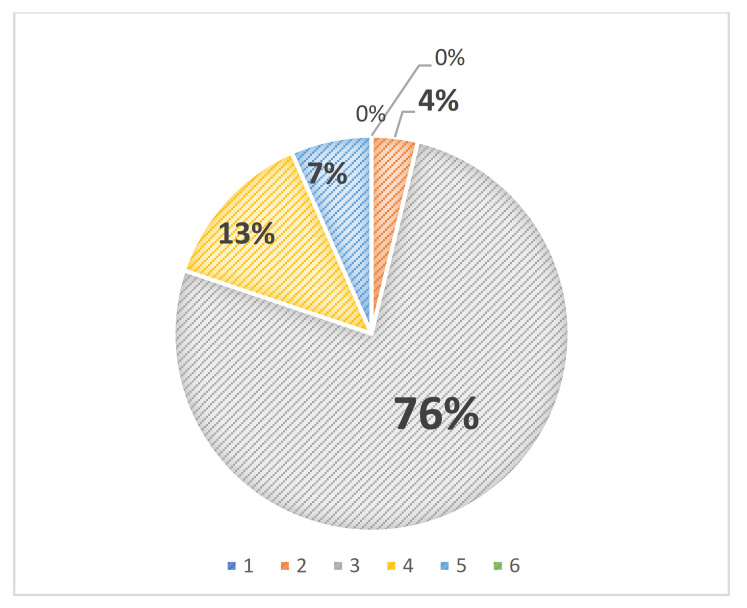
Number of course perspectives observed in the final individual essays (*n* = 106).

As already mentioned, students had to write an individual essay on a topic of choice related to pain in which at least three out of five perspectives of the course should be present. In Figure 2, the number of perspectives found in the essays is depicted. Seventy six percent of the assignments met the course requirements for the assignment. There were no essays with fewer than two or more than five course perspectives. In addition, 13% of the essays contained four perspectives, and 7% of the essays contained all five perspectives. In most cases, students met the requirements of the individual assignment. 

From Figure 2, it can be concluded that about 96% of the final essays contained three or more course perspectives. In Table 4, the percentages of the different perspectives observed in the individual essays are depicted for the different years. Both biomedical and psychological perspectives are mostly present in the essays of the year 2015–2016, while for the later years, psychological and arts perspectives were abundantly present in the essays, in 86% and 65% (2016–2017) and 86% and 78% (year 2017–2018) of the essays, respectively.

When considering both biomedical and psychological perspectives as part of a health care-oriented curriculum, dealing with perspectives such as arts, anthropology, and philosophy can be considered as students’ curiosity towards other disciplines. In fact, students were given the opportunity to explore at least one perspective outside the scope of health care. 

The top 3 topics of the final essays of Table 3 were further investigated for the frequency of the five course perspectives in these essays. As can be observed in Table 5, there is a difference in occurrence of the biomedical, psychological, and arts perspectives observed in the individual essays. Essays on song texts and (World Press) photos contained more psychological and arts perspectives, while essays on personal stories contained more biomedical and psychological perspectives compared to the anthropological and philosophical perspectives.

## 4. Discussion

### 4.1. Course Setup and Basic Psychological Needs 

Autonomy, relatedness, and competence are the basic psychological needs which foster intrinsic motivation [3,22]. From this study, it can be concluded that the course setup stimulated students’ perception of autonomy and relatedness. Autonomy in this course was not only stimulated by giving students’ freedom of choice but also via other teaching strategies. The following strategies were based upon interventions of the main teacher. Firstly, during the course, the main teacher was always in the back, coaching and challenging students by asking questions during the expert lectures, having vivid discussions with students after watching the documentary films, and sharing her personal learning objectives (teachers’ personal observations). Secondly, the teacher was always present during the expert lectures, not only to introduce the guest lecturer but also to provide students the opportunity to ask any questions on the course, course material, or assignments. The latter was intended to help students in case they wanted to discuss their problems and emotional feelings. Thirdly, during the unsupervised small group discussions the main teacher was always available by phone in case of any questions. Lastly, the main teacher also tried to interact and engage with the students by knowing their first names. Altogether, these teaching strategies might not have only stimulated students’ perception of autonomy but could have also induced feelings of relatedness and/or competency. 

Our findings on the basic psychological need satisfaction are in accordance with studies involving medical students as well as pharmacy students. Schutte et al. investigated the effect of an innovative project, the student-run clinic, on the motivation of medical students measured by Academic Motivation Scale and Intrinsic Motivation Inventory in a Dutch health care program [23]. The project combined clinical practices with high responsibility for patients and induced a learning climate where students’ basic psychological needs could be met. Schutte et al. reported satisfaction of autonomy, competence, and relatedness among medical students while following the student-run clinic. Mylrea et al. evaluated autonomy and motivation in an identity development program for pharmacy students [24]. In this program, SDT-based instruction resulted in increased levels of autonomy, thereby stimulating a shift in motivation towards intrinsic motivation. 

Our findings also support Stefanou et al.’s concept of cognitive, procedural, and organizational choices [12]. The course stimulated students’ cognitive choice by exploring multiple perspectives of pain, sharing, debating, and reflecting on their ideas on pain and individuals in pain. Moreover, students had to set their own goals within the course. As demonstrated by Vansteenkiste et al., intrinsic goal setting (e.g., for personal growth, health, community contribution, and affiliation) and autonomy-supportive activities have a synergistic effect on motivation to learn [25]. Procedural choices in the course were given to the students by means of encouraging students to gain deeper knowledge by searching for their own literature and understanding of a specific topic of interest. Although the course setup did meet, to some extent, organizational choices, for example, students could choose when to visit the museum of choice, we particularly note that our students mentioned not having a choice in picking group members. Picking your own group members can be seen as a component of organizational choice. This could be arranged in a different way in the course in the upcoming years.

In the reflective part of the final assignment, we found quotes not only on autonomy and relatedness but also on interest and relevance. Interest in a topic represents intrinsic motivation for the topic, in our case, interest in the different perspectives and/or interest in pain in general. Müller et al. also demonstrated that perceived autonomy in an autonomy-supportive environment was associated with interest and curiosity [26]. In line with Müller’s observation, pharmacy students’ interest was stimulated by the course setup and the autonomy teaching strategies. In our study, interest may positively influence students’ engagement, thereby stimulating pharmacy students’ intrinsic motivation to explore pain from different perspectives. Relevance is related to providing a rationale for learning and because of this, students understand the relevance of what they are learning for their future. This is in accordance with a study of Kramer and Kusurkar in which the authors investigated writing science blogs as a tool to stimulate students’ motivation in higher education [27]. Relevance, or “value” as it was labeled in the study of Visser et al., was seen as autonomy-supportive among medical students [28]. Understanding relevance is particularly important for students in health care professions education such as medicine, pharmacy, psychology, and nursing, as their educational programs often lead to professional practice. Further, Müller and Louw demonstrated a positive relation between perceived autonomy, intrinsic motivation, and study interest in psychology students [26]. 

Although both Kramer et al. and Schutte et al. found evidence for competence in their study, in our study, there were hardly any comments in the overall course reflection on competence [27,28]. This was surprising as students provided many comments on thinking on their own or planning the learning on their own since this is expected to have a relation to students’ competence. One explanation for the absence of quotes on competency is that, over time, students became aware that there was no strict and specific definition of the course objectives regarding the content and topic of the final individual assignments and of the final group assignment. Indeed, students had to define their own intrinsic goals (for personal development) within the topic of pain. This probably challenged pharmacy students in an optimal manner. Meeting one’s own learning objectives in a curiosity-driven learning environment may stimulate students’ interest as well as competence and thereby their motivation to learn [22]. Another explanation for the absence of quotes on competence is that students’ competence was supported by reflective assignments on documentary films. As these assignments were not graded, they could learn how to reflect in an informal manner which stimulated self-regulation, self-confidence, and competency. According to SDT, supporting competence can be facilitated by providing students with optimal challenges and performance feedback [22]. During the course, the main teacher provided some feedback on the reflective assignments if a student asked for feedback. This means that performance feedback was provided only on demand, therefore stimulating feelings of both autonomy and competence.

Based upon our results, as well as those demonstrated by Patall et al. and Stefanou et al., it can be suggested that by providing students with the freedom of choice in an autonomy-supportive learning environment, students can follow their own interest and learning path [10,12]. This may lead to engagement and relatedness towards others in the course or even towards patients in pain. In addition to this, as students may follow their own interest, they find their own relevance.

Altogether, students’ autonomy (e.g., freedom of choice) in an autonomy-supportive environment will, at least, enhance students’ relatedness, interest, and relevance, thereby enhancing students’ intrinsic motivation and motivation to learn.

### 4.2. Students’ Experiences and Interests

During the course, students found some different autonomy-supportive activities, especially unsupervised small classroom discussions and reflective writing, which was odd at the beginning. After having watched documentary films in the course, students had to write reflective essays on each of the films. These reflective writing assignments represented moments of constructive friction as most students were used to writing academic texts. Therefore, writing about feelings and emotions was new and challenging for them. At first, students tended to write about factual information from the documentary films, but gradually learned by themselves to focus on interpretation, meaning making, and sense making of the stories told in the documentary films. The reflective assignments on films were intended to relive the story and to gain an in-depth understanding not only of the content of pain but also of their own selves. Therefore, these documentary films may also stimulate students’ perspective-taking process towards others in pain and therefore may contribute to the development of empathy, which is one of the skills of health care professionals [13,29]. As demonstrated by Nunes et al. and Hojat et al., students’ empathy in health care programs declines during their education [30,31]. Nunes et al. showed that there was even a tendency for a decline in pharmacy students. However, data on empathy development and/or decline are very limited, especially in undergraduate and graduate students of pharmacy programs. It is therefore very interesting to investigate if our elective course on pain has positive effects on students’ empathy levels.

Upon expressing their feelings during class meetings as well as in their final writing assignments, students were able to discuss their dissatisfaction. Discussion about the relevance of tasks can be seen as an autonomy-supportive event. Within SDT, giving students relevance and context on why they should do such an activity would maintain or may even enhance their identified regulation [22,25]. In addition to this, discussions about the relevance of such an assignment or, in our case, the course setup are meaningful for both teacher and students, as discussed by Assor et al. [32]. Upon discussions with students on course activities, teachers themselves have to rethink and reflect on the activity, enabling them to foster students’ relevancy of the activity. As the main teacher in our course was present during class meetings and/or available on demand, students had the opportunity to discuss their dissatisfaction while the teacher was able to explain the relevancy of the activities. Therefore, these feelings of dissatisfaction were less prominent at the end of the course compared to the beginning of the course.

In our study, photos, personal stories, and song texts were popular topics for the individual final essays. These topics, together with course perspectives such as arts and philosophy, indicate that students are thinking beyond their own profession, in our case pharmacy. Thinking beyond the boundaries of their profession may have a beneficial effect on patients. Pharmacy students, especially undergraduate pharmacy students, learn about the pharmacodynamics and pharmacokinetics of drugs in the treatment of disease symptoms. They hardly encounter patients or patients’ perspective in courses. Thinking beyond the boundaries of their profession will help students to develop a patient-centered approach instead of a drug-centered approach in the treatment of diseases. Not only will this encourage them to treat patients according to the biopsychosocial model, but it will also help them to provide care in an autonomy-supportive manner. Eventually, this may lead to higher patients’ satisfaction and/or health outcomes for patients [17,18].

When looking closely at the different perspectives in the final essays, final essays based upon a personal story contained more biomedical and psychological perspectives compared to essays that dealt with song texts or (World Press) photos. Final essays based upon song texts or (World Press) photos were often related to art. This seems logical for two reasons. First, when dealing with personal stories, especially dealing with mental and/or physical health, students are likely to investigate the underlying problem from a biomedical perspective and/or psychological perspective as a way of reliving the personal story and/or a way of coping with feelings related to pain. Second, putting words in a poem-like structure (e.g., song text) or presenting photos as part of an exhibition (e.g., World Press photos) can be seen as art. 

Teaching art and/or philosophy in a science environment, and especially in one course, seems odd. Teaching art and philosophy in an autonomic supportive manner not only enables students to explore pain from an artistic and/or philosophical point of view but it also gives students enjoyment. Besides that, teaching art will provide students an opportunity to express creativity. Stimulating enjoyment as well as creativity may have a positive effect on engagement and stimulate intrinsic motivation [22]. 

There seems to be a decline over time in the percentage of essays that touched upon the biomedical perspectives upon comparing the data of the different cohorts. In addition to this decline, there seems to be an increase, and thus an interest, in the philosophical perspectives over the years. The reasons for these observations remain unclear as expert lecturers of both perspectives as well as the main teacher did not change over time. It can therefore only be explained by a shift in students’ interest in these perspectives which was probably influenced by societal problems at that time. However, it is surprising that personal stories, song texts, and photos can be handled in a philosophical manner. 

Altogether, our study suggests that it is possible to deal with the topic of pain in a multidisciplinary manner.

### 4.3. Limitations of the Study

Our study has some limitations. The qualitative data were limited because of their written nature. Conducting interviews with the students could have given us more insight into the processes underlying the impact of the autonomy support provided. Further, this study was conducted in a single institution, so the results have limited transferability. Moreover, this study did not explore a relation between the enhancement of students’ intrinsic motivation and study success. Finally, there are different ways for teachers to improve autonomy, and a more comprehensive study on the autonomy-supportive interventions in our course should be considered. Nevertheless, our findings add to the literature on autonomy-supportive strategies and their impact, especially within the topic of pain.

### 4.4. Implications of the Study and Future Directions

Our course presents a novelty in pharmacy education. Considering not only autonomy-supportive activities, such as unsupervised small group discussions, but also a complete course solely on the topic of pain is challenging. In most pharmacy curricula, pain and its specific treatment are related to the underlying disease. This approach may lead to students learning to treat a symptom of the disease with drugs while in most cases treatment of pain, especially chronic pain, can be more effective if the treatment of pain is based on an interdisciplinary approach. As reviewed by Thompson et al., design and delivery of pain education for health care professionals seem less effective if pain is approached in a traditional manner (e.g., lectures) [33]. It was even suggested to redesign curricula with an increased focus on pain. Our course is in line with such recommendations. In addition, courses and larger modules for health care professionals dealing solely with pain are limited. Therefore, our course can be inspiring for educational staff. 

Introducing learners’ autonomy into a curriculum may serve two purposes. Firstly, according to self-determination theory, learners’ autonomy plays an essential role in motivation for learning. Autonomy, together with competence and relatedness, plays an important role in everyday life situations where self-regulation is required. Secondly, being self-regulated or self-determined is pivotal for the concept of lifelong learning which has emerged as a major global educational challenge. Stimulating ownership of learning in a curriculum is expected to help students to become self-regulating learners. This is important when considering the continuous professional development and registration of Dutch health care professionals. In addition, fulfilment of basic psychological needs is also related to lifelong learning among pharmacists, as reviewed by Tjin A Tsoi et al. [34]. 

There are some further directions for this study. First, this study only shows from a teacher’s perspective what measures (e.g., autonomy-supportive teaching strategies) can be taken to enhance students’ autonomy. In order to find out if the measures are indeed autonomy-supportive from students’ perspective, the learning climate questionnaire (LCQ) can be administered. Second, data on the underlying mechanism of need satisfaction on autonomy and relatedness are missing. These can be collected by conducting semi-structured interviews with students. Third, this study does not show if enhancing the intrinsic motivation will eventually lead to a higher study success as measured by course grades compared to courses where autonomy-supportive activities are less prominent or even absent. We assume that engagement and enjoyment will positively influence students’ intrinsic motivation and thereby enhance students’ study success. We do know that all students did pass the course on a single attempt. However, we did not compare grades or percentages of students that passed/failed to other (elective) courses. Finally, the most interesting question remains as to whether students in this course gained reflective skills and attitudes so that they are able to self-assess their final essays. This will help students to become self-regulated learners and can be seen as the final step towards self-regulated learners.

## 5. Conclusions

We successfully developed a multidisciplinary course on pain in which students are exposed to different autonomy-supportive teaching strategies based on the SDT framework. The course setup met the needs of autonomy and relatedness. Although students struggled at the beginning of the course with their autonomy (e.g., freedom of choice), we can conclude that by the end of the course, students appreciated the course setup with its autonomy-supportive teaching strategies. What motivated our students to persist and overcome the discomfort of the openness needs further investigation.

## Figures and Tables

**Table 1:** Guiding questions for reflective part of the individual assignments.

Reflect in a One-Page Essay on the Content and Educational Activities of the Elective Course on Pain by Paying Attention to:
Were you able to inspire and motivate yourself to study pain from different perspectives? Which course perspectives did you find interesting? And which were not? Which lecturers were positively (or negatively) influencing you? Please explain why. Did you find the literature on the electronic learning environment informing, challenging, and/or stimulating enough? In addition to the available literature, did you search for additional information by yourself? Describe your experience on the openness in the course (e.g., less strict guidelines)? Do you have any suggestions for improvement of the course? Rate the course on a scale from 1–10. Give an explanation for your rating.

**Table 2:** Qualitative analysis—categories, codes, and quotes.

Categories	Codes and Quotes for Each Code
Interest	**Novelty in design and content** *“This course was very differently designed than our other courses.” (#001)* **Interesting content** *“The content was interesting.” (#002)* **New Way of Thinking** *“This course brought a different way of thinking to me, so I am very enthusiastic about it.” (#013)* **Challenge** *“This course let me do things that were out of my comfort zone.” (#097)* **Negative on Challenge** *“The proposed literature was without any doubt informative, but also definitely challenging, sometimes too much.” (#041)*
Autonomy	**Own ideas** *“In most cases, they were conversations about topics that everyone in the group found interesting.” (#001)* **Own structure** *“In the course, we were given a lot of freedom with regard to the topics discussed.” (#003)* **Own initiative** *“For example, we were allowed to set our own learning objectives at the start of the course.” (#009)* *“During this course I was able to inspire myself to independently delve into various aspects of pain.” (#003)* **Autonomous learning** *“I feel that the openness of this course has made me more talkative and responsible and has been able to gain more knowledge outside the pharmaceutical field.” (#105)* **Active participation** *“As with philosophy, I liked the fact that during these lectures I had to think for myself and participate.” (#010)* **Personal development** *“I really liked the openness of the course! I am actually developing myself and this helped a lot.” (#031)* **Negative choice** *“I understand the choice for assigning groups, but I think I would have preferred to choose my group members myself.” (#041)*
Relatedness	**Learning from each other** *“I always enjoy working with others, because you learn to look at certain things from different perspectives.” (#009)* **Getting to know colleagues** *“These conversations allowed us to learn a lot from each other and about each other and it ultimately ensured that we became closer as a group.” (#001)* **Good learning environment** *“In addition, I also really liked that during the tutorials my group was very open, and everyone dared to discuss everything.” (#105)* **Negative collaboration** *“This time I also experienced that the collaboration did not always go smoothly, so that many things still had to be done at the last minute and as a result half of them were unable to help with certain assignments.” (#041)*
Relevance	**Learning other perspectives** *“I learned a lot during this elective because pain has been approached from different angles.” (#020)* **For practice** *“In addition, of course he told the aspect of pain that fits within the study of pharmacy and that we as future care providers will have to deal with most.” (#016)*

**Table 3:** Different topics and their frequency observed in the final essays in the three cohorts.

Topic	2015–2016	2016–2017	2017–2018	Overall
(World Press) Photo	7	2	4	13
Sport	4	3	2	9
Historical figure	1	4	1	6
Song text	3	2	10	15
Religion	5	1	1	7
Personal stories	4	7	2	13
TV program	1	1	0	2
(Hollywood) Film	1	6	0	7
Disease	2	0	2	4
Celebrities	1	2	4	7
Documentaries	1	1	0	2
Historical event	0	2	2	4
Art	1	1	3	5
Poems	0	1	1	2
Literature	0	0	1	1
Pain in general	2	4	3	8
Total number of essays	33	37	36	106

**Table 4:** Different course perspectives (as %) observed in the final essays.

**Perspective**	**2015–2016**	**2016–2017**	**2017–2018**
Biomedical	70	57	56
Psychological	70	86	86
Arts	64	65	78
Anthropological	67	57	69
Philosophical	37	51	53
Total number of essays	33	37	36

**Table 5:** Frequency of the course perspectives observed in the top 3 final essays from all cohorts (2015–2018).

Perspective	Song Text (*n* = 15)	Personal Stories (*n* = 13)	(World Press) Photo (*n* = 13)
Biomedical	7	12	6
Psychological	12	10	9
Arts	13	5	10
Anthropological	8	6	8
Philosophical	8	8	7

## Data Availability

The data presented in this study are available on request from the corresponding author. The data, although anonymized, are not publicly available due to the personal nature of student narratives.
